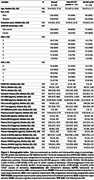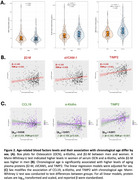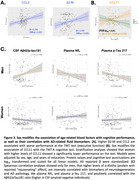# Sex‐specific associations between age‐related blood proteins, cognition, and Alzheimer's biomarkers

**DOI:** 10.1002/alz70856_107568

**Published:** 2026-01-09

**Authors:** Luis Felipe Hernández‐Villamizar, Luisa Sophie Braun‐Wohlfahrt, Greta Garcia‐Escobar, Marina De Diego‐Osaba, Helena Blasco‐Forniés, Paula Ortiz‐Romero, Esther Jiménez‐Moyano, José Contador, Isabel Estragués‐Gázquez, Rosa Maria Manero‐Borràs, Irene Navalpotro, Oriol Grau‐Rivera, Aida Fernández‐Lebrero, Marta del Campo, Albert Puig‐Pijoan, Federica Anastasi, Marc Suarez‐Calvet

**Affiliations:** ^1^ Barcelonaβeta Brain Research Center (BBRC), Pasqual Maragall Foundation, Barcelona, Spain; ^2^ Hospital del Mar Research Institute (IMIM), Barcelona, Spain; ^3^ Departament de Bioquímica i Biologia Molecular, Universitat Autònoma de Barcelona (UAB), Barcelona, Catalonia, Spain; ^4^ University of Gothenburg, Gothenburg, Sweden; ^5^ Servei de Neurologia, Hospital del Mar, Barcelona, Spain; ^6^ Department of Medicine and Life Sciences, Universitat Pompeu Fabra (UPF), Barcelona, Spain; ^7^ Centro de Investigación Biomédica en Red de Fragilidad y Envejecimiento Saludable (CIBERFES), Instituto de Salud Carlos III, Barcelona, Spain; ^8^ Centre for Genomic Regulation (CRG), Barcelona Institute of Science and Technology (BIST), Barcelona, Spain

## Abstract

**Background:**

Peripheral blood factors influence brain aging in animal models, but their role in humans, particularly in age‐related neurodegenerative diseases like Alzheimer's disease (AD), remains unclear. We investigated whether these blood factors are associated with AD‐related biomarkers and cognitive performance in cognitively impaired patients.

**Method:**

This cross‐sectional study measured 10 age‐related blood proteins in 366 participants from the BIODEGMAR cohort (Hospital del Mar, Barcelona), with cognitive impairment (median age of 74.4 years, IQR:70.0‐77.5; 57% women; 44% *APOE*‐ε4 carriers; 62,8% were CSF amyloid‐positive; Figure 1). Blood proteins and AD‐related biomarkers were quantified using ELISAs, MSD, Simoa or Lumipulse platforms. Linear regression models assessed associations with chronological age, and cognitive performance, considering sex and amyloid status as potential modifiers. Benjamini‐Hochberg false discovery rate (FDR) correction was applied to adjust for multiple comparisons.

**Result:**

Osteocalcin and a‐Klotho in blood were higher in women, while β2‐microglobulin was higher in men (Figure 2A). Older chronological age was associated with higher β2‐microglobulin (β=+0.189, FDR‐p=0.002), TIMP2 (β=+0.240, FDR‐p=<0.001), and sVCAM1 (β=+0.160, FDR‐p=0.008) (Figure 2B). Sex modified associations of CCL19, α‐Klotho, and TIMP2 with chronological age (Figure 2C); in men, older age was associated with lower CCL19 (β=‐0.204, FDR‐p=0.027) and α‐Klotho (β=‐0.168, *p* = 0.040), but higher TIMP2 (β=0.382, FDR‐p=<0.001). Higher CCL2 and β2‐microglobulin were associated with worse cognitive performance in the overall sample (Figure 3A), while higher CCL11 was associated with poorer executive function in women only (β=+0.321, FDR‐p=0.030) (Figure 3B). Finally, higher α‐Klotho correlated with lower plasma *p*‐tau217, NfL, and with higher CSF Aβ42/p‐tau181 in men only (Figure 3C).

**Conclusion:**

Sex modulates the relationship between age‐related blood factors, and chronological age, cognition, and AD‐related biomarkers. α‐Klotho was linked to lower AD pathology only in men, while CCL11 was associated with worse executive function in women. These findings highlight sex‐specific biological pathways in aging and neurodegeneration.